# Prenatal mTOR Inhibitors in Tuberous Sclerosis Complex: Current Insights and Future Directions

**DOI:** 10.3390/jcm13216335

**Published:** 2024-10-23

**Authors:** Giacomo Racioppi, Martina Proietti Checchi, Giorgia Sforza, Alessandra Voci, Luigi Mazzone, Massimiliano Valeriani, Romina Moavero

**Affiliations:** 1 Residency School of Pediatrics, University of Rome Tor Vergata, 00133 Rome, Italy; 2 Academy of Pediatrics, Bambino Gesù Children’s Hospital, IRCCS, 00165 Rome, Italy; 3 Developmental Neurology Unit, Bambino Gesù Children’s Hospital, IRCCS, 00165 Rome, Italy; martina.proietti@opbg.net (M.P.C.); giorgia.sforza@opbg.net (G.S.); alessandra.voci@opbg.net (A.V.); valeriani@opbg.net (M.V.); romina.moavero@opbg.net (R.M.); 4 Child Neurology and Psychiatry Unit, Systems Medicine Department, Tor Vergata University of Rome, 00133 Rome, Italy; luigi.mazzone@uniroma2.it; 5 Center for Sensory Motor Interaction, Aalborg University, DK-9220 Aalborg, Denmark

**Keywords:** mTOR Inhibitors, rapamycin, sirolimus, tuberous sclerosis, TSC1, TSC2, prenatal, foetal, pregnancy, rhabdomyoma

## Abstract

**Background**: Tuberous sclerosis complex (TSC) can present prenatally, often with cardiac rhabdomyomas, which, if large, may cause complications such as hydrops fetalis and reduced cardiac output. Prenatal treatment of these lesions with mTOR inhibitors, approved for other TSC manifestations, is under investigation. We hypothesize that mTOR inhibitors could help manage or prevent other TSC-related conditions, particularly neurological issues like epilepsy and CNS lesions, potentially improving neurodevelopmental outcomes. However, the safety of prenatal mTOR treatment remains a concern, especially for foetal development, and limited data are available on neurological outcomes. **Methods**: We conducted a literature review using PubMed, EMBASE, and Cochrane CENTRAL, focusing on studies involving mTOR inhibitors for prenatal TSC management. The search included case reports and series involving pregnant women diagnosed with TSC or early manifestations like cardiac rhabdomyomas. Keywords included “mTOR Inhibitor”, “Rapamycin”, “tuberous sclerosis complex”, “prenatal”, and “rhabdomyoma”. **Results**: Three prenatal mouse studies and eight papers reporting on ten pregnant women treated with mTOR inhibitors were identified. **Conclusions**: The literature confirms that prenatal mTOR inhibitors may reduce cardiac rhabdomyomas. However, further studies are needed to explore their broader potential, particularly in preventing neurological complications, while carefully considering their impact on intrauterine growth and neurodevelopment.

## 1. Introduction

Tuberous sclerosis complex (TSC) is a rare autosomal dominant multisystem disease, with a prevalence of approximately 1 in 6000 live births [1]. It is characterized by the development of benign tumours, known as hamartomas, in various organs, including the brain, kidneys, skin, heart, and lungs [2]. TSC is caused by inactivating mutations in either the *TSC1* or *TSC2* genes, which encode the proteins hamartin and tuberin, respectively. These two proteins form a heterodimeric complex that inhibits the GTPase Rheb, thereby suppressing the mammalian target of rapamycin complex 1 (mTORC1) [3]. When this inhibitory pathway is disrupted, mTORC1 becomes hyperactive, driving the abnormal growth of cells and leading to the formation of TSC-associated lesions [4,5]. Moreover, mTORC1 hyperactivation plays a pivotal role in shaping the neurological manifestations of TSC, which are among the most debilitating aspects of the disease [6].

While TSC affects multiple organ systems, neurological involvement has the most significant impact on patient morbidity and mortality [7]. Neurological lesions include subependymal nodules, subependymal giant cell astrocytomas, radial migration lines, and cortical tubers. Historically, cortical tubers have been regarded as the primary epileptogenic foci; however, growing evidence suggests that the “normal appearing perituberal cortex” may also be a source of seizures [8]. The age at seizure onset and the timing of the appearance of epileptiform abnormalities on the electroencephalogram (EEG), as well as their localization, may correspond to the placement of cortical tubers detected through magnetic resonance imaging (MRI). These events often align with the functional maturation of the cortex, with seizures appearing earlier in the temporo-occipital regions compared to the frontal areas [9].

Histologically, cortical tubers and “normal-appearing perituberal cortex” exhibit a spectrum of abnormalities, including the loss of the typical six-layered cortical structure and the presence of aberrant cell types such as dysplastic neurons, giant cells, and abnormally shaped astrocytes. These dysplastic neurons and astrocytes exhibit altered expression of gamma-aminobutyric acid (GABA) transporters and glutamate receptors [10]. This imbalance in neurotransmission, characterized by decreased GABA-mediated inhibition and increased glutamate-driven excitation, is thought to underlie epileptogenesis in TSC. The deficiency of GABAergic interneurons may also contribute to the early onset and severity of seizures in TSC patients [11]. Moreover, the disruption of GABAergic neurotransmission is believed to serve as a potential neurobiological link between epilepsy and autism spectrum disorder (ASD), which is prevalent in TSC [12]. 

Among the neurological challenges posed by TSC, pharmacoresistant epilepsy is particularly prominent, affecting approximately two-thirds of patients. Additionally, around half of the affected individuals experience varying degrees of intellectual disability, with a similar proportion being diagnosed with autism spectrum disorder [13,14]. These neurological deficits not only complicate clinical management but also have profound implications for the patient’s overall quality of life, especially given the long-term cognitive and behavioural consequences [15].

One of the most significant challenges is addressing TSC-associated neuropsychiatric disorders, also known as TAND, which are reported in 90% of affected individuals, starting in childhood [16]. These disabilities appear to have age-related presentations, depending on different developmental stages, and require a broad perspective and a multidisciplinary approach. This involves professionals such as psychiatrists, psychologists, occupational therapists, and educators, with individualized care tailored to each stage of life [17]. TAND has been studied more extensively in terms of its psychiatric aspects, rather than its social and behavioural dimensions, despite the well-known association between TSC and autism spectrum disorder (ASD), with TSC being one of the leading single-gene disorders causing ASD [18]. A more precise assessment of clinical features, such as genetic mutations, MRI findings, and epilepsy history, has only recently been proposed as a way to predict the development of ASD, emphasizing the critical role these factors play in managing the neurocognitive burden [19]. 

In recent years, there has been a growing focus on early and aggressive treatment of epilepsy in TSC, driven by the recognition that recurrent seizures, especially in early life, can have detrimental effects on neurodevelopment [20]. Early treatment strategies are designed to mitigate the long-term impact of these seizures on cognitive, language, and motor development [21]. As part of this shift, preventive treatment with vigabatrin (VGB) is now widely recommended. This treatment is typically initiated as soon as epileptiform abnormalities are detected, even before the clinical manifestation of seizures [22]. VGB is a GABA transaminase inhibitor that can completely halt spasms in 95% of infants with TSC. It is recommended as the first-line treatment for early-onset seizures, including both focal seizures and infantile spasms (IS) [23,24]. This drug is highly effective for this specific condition, as it increases GABA availability in the tuberal area, thereby preventing the spread of paroxysmal activity beyond the cortical dysplasia [25]. However, this is not the sole reason for VGB’s unique efficacy in TSC-related epilepsy. Unlike other GABA-enhancing drugs such as barbiturates and benzodiazepines, VGB has been shown to inhibit mTOR overactivation, which may contribute to its effectiveness [26]. 

However, results from a recent multicentre study suggest that although this treatment strategy is quite effective in reducing the onset and/or severity of epilepsy, there is no clear effect on neurodevelopmental outcomes [27]. Nevertheless, more recent studies aimed to demonstrate that individuals treated with sirolimus or vigabatrin as preventive therapy before symptom onset will perform better in terms of cognitive, language, and motor development compared to those who did not receive treatment or were treated after symptom onset. This raises the possibility that antiseizure medication alone may be insufficient to alter the disease’s neurodevelopmental trajectory [28]. 

In recent decades, significant advancements have been made in the treatment of this complex pathology, especially with the introduction of drugs specifically inhibiting the mTOR complex. These drugs have been approved for the treatment of TSC-related SEGAs, epilepsy, and renal angiomyolipomas, offering a more targeted therapeutic approach that addresses the underlying molecular pathology.

mTOR inhibitors represent a relatively new pharmacological class, firstly born with rapamycin, a natural substance with antifungal properties isolated from Streptomyces species. In addition, it also showed anticancer activity, leading to the current clinical use [29]. Sirolimus is a biochemical, functional form of rapamycin derived from the expression of the specific molecular domain (FKBP12 and FKBP51) that determines the pharmacological effect. Everolimus is a modified drug derived from sirolimus [30]. Rapamycin and its derivatives bind to the FKBP12 (FRB-FK506 binding protein 12), forming a protein complex. This complex then binds to mTOR through its FRB (FKBP–rapamycin binding) domain, inhibiting mTOR and its downstream effectors. As a result, cells are arrested in the G1 phase of the cell cycle, leading to apoptosis [31]. Clinical studies on TSC-related manifestations primarily focus on everolimus, the only mTOR inhibitor approved for treating this complex disease. However, few studies compare everolimus with other mTOR inhibitors. Like earlier compounds, everolimus is biochemically active without modification and is administered orally once daily. Compared to sirolimus, it has several pharmacokinetic differences, including better absorption, higher oral bioavailability, and faster steady state and clearance, while maintaining similar pharmacodynamics [32].

Animal studies suggest that preventive treatment with mTOR inhibitors could not only prevent seizure onset but also reverse certain social deficits observed in mouse models of TSC [33,34,35]. However, while case reports indicate potential behavioural benefits from mTOR inhibitors in humans, larger studies have yet to conclusively demonstrate their efficacy in reversing neuropsychological and cognitive deficits [36,37,38,39]. This may be due, in part, to the age of patients included in these studies, as pharmacological interventions in older children may be less effective at reversing years of neural dysfunction. As a result, scientific efforts are now concentrated on identifying the critical time window during which therapeutic interventions might yield the greatest benefit.

TSC is increasingly being diagnosed prenatally, often following the detection of cardiac rhabdomyomas—benign tumours that serve as the earliest sign of the disease. These tumours, which are associated with TSC in up to 96% of cases involving multiple lesions, are typically asymptomatic and benign [40]. Generally, the literature agrees that cardiac rhabdomyomas grow most significantly during the second and third trimesters, then slow down after 32 weeks and toward the end of pregnancy. This could be due to the influence of estrogenic hormones during pregnancy on tumour growth [41]. However, most rhabdomyomas have a favourable course, showing continuous regression in size and complete resolution in over 80% of cases by early childhood, with no further cardiac involvement or major cardiovascular complications [42]. The average time for shrinkage is well established to be within the first six years of life, possibly due to apoptosis, though the exact mechanism remains unknown. In females, particularly during adolescence, symptomatic or asymptomatic growth or the appearance of new rhabdomyomas may occur, possibly linked to oestrogen’s proliferative effect on cardiac cells [43]. Additionally, genetic variability has been suggested as a possible explanation for differing growth patterns, particularly in rare cases where large masses develop very early. However, no data currently support a genetic hypothesis in the pathogenesis of rhabdomyomas in TSC [44].

The ability of mTOR inhibitors to reduce rhabdomyoma size after birth is well established, as demonstrated in numerous case series. These inhibitors are now considered a viable alternative to invasive therapies, with a high success rate [45,46]. On average, a significant reduction in the size of cardiac masses, or even complete regression, is reported within 2–3 months, with some cases showing improvement in under a month [47]. Serial echocardiographs are used to track the progressive effectiveness of the therapy, though a rebound in tumour size may occur after rapid discontinuation of mTOR inhibitors, necessitating close follow-up, even after complete regression of cardiac lesions [48]. However, when rhabdomyomas are large enough to obstruct cardiac flow, they can cause serious complications such as hydrops fetalis and reduced cardiac output, which may lead to prenatal or early postnatal death [49]. In some cases, the detection of large lesions has prompted attempts at prenatal treatment with mTOR inhibitors, both in animal models and in pregnant women. Despite the promise of this approach, very little is known about the long-term effects of prenatal mTOR inhibition. The aim of this paper is to review the existing literature on prenatal treatment with mTOR inhibitors, with a focus on potential long-term and systemic effects, as well as possible adverse outcomes.

## 2. Materials and Methods

We conducted a comprehensive search of the PubMed, EMBASE, and Cochrane Central Register of Controlled Trials (CENTRAL) databases to identify relevant studies on the use of mTOR inhibitors in the prenatal management of tuberous sclerosis complex (TSC). The search strategy was designed to capture all pertinent articles using a combination of keywords and MeSH terms. The following search terms were employed: “mTOR inhibitor” OR “everolimus” OR “rapamycin” AND “prenatal” OR “foetal” OR “pregnancy” AND “tuberous sclerosis complex” OR “tuberous sclerosis” OR “TSC”. These terms were chosen to ensure a broad, yet focused retrieval of studies related to the prenatal application of mTOR inhibitors in TSC-affected pregnancies.

We applied no restrictions regarding publication date to ensure that older, potentially seminal studies were included, while focusing primarily on peer-reviewed articles published in English. In addition, we manually screened the references of the retrieved studies to identify any further relevant articles that may not have been captured by our initial search. Conference abstracts, case reports, and reviews were also considered if they provided novel data or perspectives relevant to prenatal mTOR inhibition in TSC. Where necessary, duplicate entries across the databases were removed prior to the analysis.

The final dataset was filtered based on predefined inclusion and exclusion criteria, which focused on studies reporting the effects of prenatal mTOR inhibition on foetal outcomes, long-term neurodevelopment, or systemic effects in either animal models or human cases.

## 3. Results

### 3.1. Animal Models

We found three mouse model studies aimed at investigating the potential effects of prenatal treatment with mTOR inhibitors, particularly on its psychomotor and behavioural development. Data are summarized in Table 1.

In the first study by Anderl et al. [50], the authors studied a TSC1-mutant mouse model characterized by brain enlargement, mTOR hyperactivation, and neonatal death due to neurobehavioral effects, such as poor mother–pup interaction, with limited milk suckling and consequent hypoglycaemia. They demonstrated that a single dose of prenatal rapamycin led to a dramatic improvement in the survival of the pups. This treatment was supplemented with further administration of rapamycin starting at postnatal day 8 and continued at gradually increasing doses. Although this treatment prolonged survival (up to 40 days), the mice showed low birth weights, associated with poor weight gain, developmental delay and neurological symptoms such as tremor, delayed eye opening beyond age 3 weeks, and the “Straub tail” phenomenon (tail spasms, resembling those typically observed after opioid administration). These symptoms did not appear in the prenatally treated wildtype, suggesting a reversal in mTORC1 activity, reducing the pathway in favour of mTORC2, and consequent hyperactivation of Akt, which contributes to poor neurocognitive development.

Way et al. [51] conducted a three-arm study that included prenatal therapy, a combined prenatal and postnatal therapy scheme, and postnatal therapy only, all using rapamycin. To minimize the risk of intrauterine growth restriction (IUGR) and postnatal growth issues, the lowest possible dose was administered. An increase in mean survival was observed across all three groups. The combined and postnatal groups were indistinguishable from healthy controls at birth but began to experience mortality around 40 days after birth, underscoring the necessity for continuous treatment. Prenatal therapy alone did not definitively block the mTORC1 pathway and thus did not alter the natural course of the disease or affect mortality. However, histological examination revealed a significant reduction in cellular hypertrophy and hyperplasia in all three groups. Clinically, the combined treatment showed the greatest neurodevelopmental recovery by blocking the altered prenatal cortical migration. Nonetheless, animals treated with the combined therapy did not perform as well as postnatally treated animals in learning and memory tasks.

A subsequent study [52] focused on the clinical effects of rapamycin during early development and adulthood, not specifically in TSC-mutant mice. In this study, pregnant wildtype mice received a single intraperitoneal dose of rapamycin at a gestational age of 16.5 days, which corresponds to the second trimester in human gestation. This timing was chosen to minimize the risk of interfering with organogenesis if administered too early and to avoid birth complications if administered too late. The results showed that the treated mice, monitored blindly from short-term follow-up into adulthood, exhibited an anxious phenotype and delays in psychomotor development stages. However, no alterations were observed in their development of relational or social skills.

**Table 1:** Animal models: prenatal treatment with mTOR inhibitors in TSC1- or TSC2-knockout and wildtype mice.

Nr.	Reference	Mutant Gene	GA at Therapy	Drug	Route of Administration	Dosage	Therapy after Birth	Age of Follow-Up	Follow-Up
1	Tsai ’13 [52]	Wildtype	E16.5	SIR	IP	1 mg/kg once	no	4–7 weeks	Delay in sensorimotor and motor milestones, anxious phenotype, no effects on social behaviour
2	Way ’12 [51]	TSC2	E12.5-P0 (prenatal) E12.5-P21 (combined) P0-P21 (postnatal)	SIR	IP	0.1 mg/kg/day	Yes—IP (only combined and postnatal group)	P120	Reduction of survival, less effectiveness in restoring neurodevelopment Long-term memory (consolidation) disfunction Best performance in hippocampus-dependent memory tasks
3	Anderl ‘11 [50]	TSC1	E15-17	SIR	SC	1 mg/kg once	Yes—IP (P8–P19: 1 mg/kg, every 3–4 days; >P21: 3 mg/kg, 3 times/week)	P40	Reduction in the weight of the newborn, developmental delay, neurological symptoms

[GA: gestational age; E: embryonal (prenatal) day; P: postnatal day; SIR: sirolimus; IP: intraperitoneally; SC: subcutaneous].

### 3.2. Clinical Studies

Our search identified eight papers [53,54,55,56,57,58,59,60] comprising case reports and case series, detailing ten pregnant women treated with mTOR inhibitors following a prenatal TSC diagnosis. In all cases, the diagnosis was made after the detection of cardiac rhabdomyomas, which were also the indication for treatment. These rhabdomyomas were detected in only two cases where the mothers had a prior diagnosis of TSC. In the remaining cases, the rhabdomyomas were incidentally discovered during the first morphological screening ultrasound. Genetic testing was performed either in utero following clinical suspicion or after birth. In one case, a prenatal diagnosis of TSC was made based on foetal MRI, which showed CNS involvement. More details are listed in Table 2.

Sirolimus was the most frequently administered drug, with everolimus used in only one case. Therapy typically began between the end of the second and the beginning of the third trimesters, ranging from a minimum of 23 to 36 weeks of gestation. The treatment continued for varying periods, with regular clinical evaluations and blood tests every 1–2 weeks and foetal echocardiographic assessments every 2 weeks. The average dosage was progressively adjusted based on blood levels, which were usually maintained between 10 and 15 ng/ml. In almost all cases, therapy was continued until birth, though in some instances, it was discontinued one week before the scheduled birth or even earlier. Achieving the desired blood levels corresponded with a gradual reduction in the size of intracardiac lesions, with one case showing total disappearance. Except for one case ([57]) where preterm birth was planned due to intrauterine growth restriction, no adverse effects on the foetus were observed. In the remaining cases, pregnancies progressed favourably for both the foetus and the mother, with full-term births and no perinatal issues.

Only one newborn received immediate postnatal therapy with an mTOR inhibitor, specifically everolimus [53], due to its positive effects on the size of the known rhabdomyoma. Follow-up until 13 months of age documented developmental delay, electroencephalogram (EEG) abnormalities, leading to the initiation of prophylactic therapy with Vigabatrin, and recurrent major infections, including viral pneumonia. Consequently, clinicians discontinued everolimus therapy at 20 months.

Two other patients started therapy at 4 days and 2 months of age to prevent tumour progression or because the tumour progressed after an attempt to discontinue therapy. Follow-up at 36 months and 9 months, respectively, showed no developmental alterations or EEG abnormalities.

Among the other newborns who did not undergo postnatal therapy, one patient exhibited EEG abnormalities at follow-up, prompting the initiation of antiepileptic therapy with phenobarbital. Additionally, delayed speech was observed in three other cases (at 16, 21, and 24 months).

All patients studied exhibited normal postnatal growth during the observed period, including the newborn who experienced intrauterine growth restriction.

## 4. Discussion

Tuberous sclerosis complex (TSC) remains a challenging genetic disorder, particularly given the wide range of organ systems it affects and the significant neurological complications it introduces. The underlying pathophysiology of TSC revolves around mutations in the TSC1 or TSC2 genes, leading to the dysregulation of the mTORC1 pathway and resulting in abnormal cell proliferation and tumour growth. Among these manifestations, cardiac rhabdomyomas are often the first detectable sign of TSC, and their presence in the prenatal stage offers a window of opportunity for early intervention.

The use of mTOR inhibitors as a therapeutic option in TSC is well-established in postnatal settings, with proven efficacy in reducing tumour size and controlling refractory epilepsy in some cases. However, prenatal treatment with mTOR inhibitors remains largely experimental, and as shown in our review, the long-term impact of such interventions—especially concerning neurodevelopment—requires much more scrutiny. The safety and efficacy of mTOR inhibitors during pregnancy are of particular concern, given their teratogenic classification in animal studies, yet the limited human data available suggest that these drugs may be safe, or at least not overtly harmful to foetal development [61]. 

Despite promising results, our review highlights several gaps and challenges. Firstly, although prenatal mTOR inhibition has been effective in reducing the size of cardiac rhabdomyomas, it remains unclear if this effect translates into improved long-term outcomes for the child, particularly in terms of neurological development. Cardiac symptoms in newborns can be life-threatening, and reducing tumour burden in utero is a significant achievement, potentially preventing prenatal complications such as hydrops fetalis. However, as our findings suggest, the full neurological impact of TSC, including the high prevalence of epilepsy and autism spectrum disorder (ASD), may not be significantly altered by such interventions.

This brings us to an important consideration: while the early treatment of TSC-related tumours is clearly advantageous, mTOR inhibitors may not address the full spectrum of TSC neurodevelopmental challenges. Epileptogenesis, in particular, remains a complex process that likely begins before birth and involves not just the presence of cortical tubers but also disruptions in cortical migration and synaptic function. The observation of EEG abnormalities in some children treated prenatally raises the question of whether mTOR inhibition, particularly when administered late in gestation, can adequately prevent or mitigate the development of epilepsy and other neurological deficits. Furthermore, there is still insufficient data to determine if sirolimus and everolimus have different effects during this specific period of life.

Moreover, the timing of mTOR inhibitor administration appears critical. Animal models suggest that early prenatal treatment may offer benefits in terms of neurocognitive recovery, but these effects are not uniformly observed in human studies. Postnatal supplementation with mTOR inhibitors may still be necessary, as seen in some cases where continuous therapy was required to maintain tumour reduction or prevent postnatal disease progression. However, the limited cognitive and behavioural improvements observed in some mouse models raise concerns about whether prenatal interventions alone can significantly alter the disease trajectory, particularly when initiated later in pregnancy.

Additionally, the dose-related complications noted in animal studies, including intrauterine growth restriction (IUGR) and developmental delays, call for careful monitoring of the dosage and timing of treatment in human pregnancies. While no major congenital abnormalities have been reported in the cases we reviewed, language delays and developmental abnormalities were noted in some children, which could either be a reflection of the underlying TSC pathology or a subtle effect of mTOR inhibitor exposure.

Finally, the long-term neurological effects of prenatal mTOR inhibition remain largely unexplored. While the immediate postnatal outcomes have been mostly favourable in terms of growth and tumour control, the neurodevelopmental trajectory of these children over time needs further investigation. Given the high rate of neuropsychiatric manifestations in TSC, the lack of detailed neurodevelopmental follow-up in the reported cases represents a significant gap in the literature.

## 5. Conclusions

In conclusion, while the data from animal models and early clinical case reports provide encouraging evidence that prenatal treatment with mTOR inhibitors can reduce the size of cardiac rhabdomyomas and potentially improve immediate outcomes, there remains substantial uncertainty regarding the long-term effects of such interventions on neurodevelopment. The small number of cases, combined with the heterogeneity in treatment protocols and follow-up periods, makes it difficult to draw definitive conclusions.

The discrepancies between animal model outcomes and human clinical experiences underscore the complexity of translating preclinical findings into therapeutic interventions. It is clear that mTOR inhibitors hold promise as part of the prenatal management of TSC, but additional studies are urgently needed to fully assess their impact on neurological outcomes, including seizure onset and cognitive development.

Future research should prioritize longitudinal studies with comprehensive follow-up into childhood and beyond, with a particular focus on neurodevelopmental milestones, epileptogenesis, and behavioural outcomes. Multicentre clinical trials could help establish standardized treatment protocols and clarify the potential risks and benefits of prenatal mTOR inhibition. Only through such rigorous studies can we better understand whether early intervention with mTOR inhibitors can truly alter the natural history of TSC, improving both the neurological and overall quality of life for affected individuals.

For now, prenatal mTOR inhibition remains an experimental but promising avenue for treating TSC. Continued efforts to refine this approach, ensuring its safety and efficacy, will be critical in shaping the future of prenatal care for this complex disease.

## Figures and Tables

**Table 2:** Clinical studies: human cases of prenatal treatment in utero with mTOR inhibitors.

N	Reference	GA at Therapy Start–Stop (Weeks)	GA at Birth (Weeks)	Genetics	Drug	Dosage (mg)	Maternal Bood Level (ng/mL)	Adverse Prenatal Events	Postnatal Therapy	Adverse Postnatal Events	Last Follow-Up (Months)	Epileptic Seizures	EEG Abnormalities	Psychomotor Delay
**1**	Carsten-Will, ’23 [53]	27–38	39 + 1	*TSC2* (familial, father)	SIR	4	8.4–9.9	NO	EVE (to 20 months)	Recurrent infections: viral pneumonia stopped therapy	13	N	Y	Y
**2**	Dagge, ’22 [54]	26–birth	39	NA	SIR	4–10	13.8	NO	NO	/	/	/	/	/
**3**	Cavalheiro, ’21 [55]	/	39	NA	EVE	10	8.4	NO	EVE (at 4 days)	/	36	N	N	N
**4**	Ebrahimi-Fakhari, ’21 [56]	35 + 2–birth 32 + 3–birth 34–birth	39 + 1 36 + 6 38 + 6	NA	SIR	3 3 4 + 2	6.1 3.7 10.85 ± 1.26	/	NO	/	24 21 16	N	N	Y (delayed speech)
**5**	Pluym, ’19 [57]	28–35	36	*TSC2* (de novo)	SIR	6–10	11.6–18.6	IUGR	NO	/	6	N	N	N
**6**	Vachon-Marceau, ’19 [58]	31 + 4–36	39	*TSC2* (de novo)	SIR	5–8	TARGET (not specified)	NO	NO	/	/	N	Y	N
**7**	Park, ’19 [59]	23–birth	39	*TSC2* (familial, mother)	SIR	4–12	12.1	NO	NO	/	/	/	/	/
**8**	Barnes, ‘18 [60]	30–birth	36	*TSC1*	SIR	/	/	NO	SIR (at 2 months)	/	9	N	N	N

[GA: gestational age; NA: not available; SIR: sirolimus; EVE: everolimus; EEG: electroencephalography].
